# Egg Yolk IgY: A Novel Trend of Feed Additives to Limit Drugs and to Improve Poultry Meat Quality

**DOI:** 10.3389/fvets.2020.00350

**Published:** 2020-07-14

**Authors:** Mohamed A. Hussein, Ibrahim F. Rehan, Ahmed F. Rehan, Nesreen Z. Eleiwa, Mootaz A. M. Abdel-Rahman, Sohaila G. Fahmy, Ahmed S. Ahmed, Mohammed Youssef, Hassan M. Diab, Gaber E. Batiha, Sara T. Alrashood, Haseeb A. Khan, Obeid Shanab, Eslam Ahmed, Hamdy Hassan, Asmaa Elnagar, Amr Elkelish, Abd El-Latif Hesham, Mohamed A. Maky

**Affiliations:** ^1^Department of Food Control, Faculty of Veterinary Medicine, Zagazig University, Zagazig, Egypt; ^2^Department of Husbandry and Development of Animal Wealth, Faculty of Veterinary Medicine, Menofia University, Shebin Alkom, Egypt; ^3^Department of Food Hygiene, Agricultural Research Center, Animal Health Research Institute, Giza, Egypt; ^4^Department of Behavior, Management and Development of Animal Wealth, Faculty of Veterinary Medicine, Minia University, El-Minia, Egypt; ^5^Department of Animal Behaviour and Management, Faculty of Veterinary Medicine, South Valley University, Qena, Egypt; ^6^Department of Food Hygiene and Control (Milk Hygiene), Faculty of Veterinary Medicine, South Valley University, Qena, Egypt; ^7^Department of Animal Physiology, Faculty of Veterinary Medicine, South Valley University, Qena, Egypt; ^8^Department of Animal and Poultry Health and Environment, Faculty of Veterinary Medicine, South Valley University, Qena, Egypt; ^9^Department of Pharmacology and Therapeutics, Faculty of Veterinary Medicine, Damanhour University, Damanhour, Egypt; ^10^Department of Pharmaceutical Chemistry, College of Pharmacy, King Saud University, Riyadh, Saudi Arabia; ^11^Department of Biochemistry, College of Science, King Saud University, Riyadh, Saudi Arabia; ^12^Department of Biochemistry, Faculty of Veterinary Medicine, South Valley University, Qena, Egypt; ^13^Department of Animal Production, Faculty of Agriculture, South Valley University, Qena, Egypt; ^14^Department of Biochemistry, Faculty of Veterinary Medicine, Zagazig University, Zagazig, Egypt; ^15^Department of Botany, Faculty of Science, Suez Canal University, Ismailia, Egypt; ^16^Department of Genetics, Faculty of Agriculture, Beni-Suef University, Beni-Suef, Egypt; ^17^Department of Food Hygiene and Control (Meat Hygiene), Faculty of Veterinary Medicine, South Valley University, Qena, Egypt

**Keywords:** feed additives, hygiene, egg yolk IgY, meat quality, probiotic

## Abstract

Drugs that are commonly used in poultry farms can potentially cause a detrimental effect on meat consumers as a result of chemical residues. Therefore, seeking a natural alternative is crucial for the health of the consumers. The egg yolk immunoglobulin Y (IgY) is a promising natural replacement for antibiotics in the broilers' diet. There is a scarce focus on the influence of probiotics and IgY on the quality and the nutritive values of broiler meat and whether it can efficiently displace the anti-microbial power of antibiotics. Herein we used 40 Ross chicks (1.2 ± 0.43 days old) and separated them into four groups with variant feed additives (basal diet “control,” probiotic, IgY, and probiotic + IgY). Our findings showed that the combination of probiotic and IgY supplementation enhanced the carcass quality traits and decreased the pH values that could retard spoilage due to bacteria and improve shelf life and meat quality. The same group also achieved a significant reduction in thiobarbituric acid value, indicating an improvement of meat quality. Moreover, color, shear force, water holding capacity, and cooking loss were most acceptable in broiler meat supplemented with IgY, which confirmed the highest carcass quality. Notably, the weight gain in the combination group has been greatly increased. Also, the protein percentage was the highest (22.26 ± 0.29, *P* < 0.001) in this combined supplementation group, which revealed the highest nutritive values. *Staphylococcus aureus* and *Escherichia coli* could not be detected in the meat of the probiotics group and/or in the combined treatment group. Interestingly, the IgY group showed an evidence of the killing power (log colony-forming units per milliliter) of *S. aureus* and *Listeria monocytogenes* at 1,500 μg/ml. Our findings, *in vitro* as well as *in vivo*, revealed that the combination group had antimicrobial bioactivity and enhanced the chickens' immunity. Therefore, IgY, a novel trend of feed additives, can be used to limit drugs. Additionally, the mortality percentage recorded was zero in all groups that received feed supplementation, while the combination group reached the best financial advantages. We concluded that feeding IgY powder with probiotic is a frontier to improve the productivity, immunity, and meat quality of broilers.

## Introduction

The administration of antibiotics for chickens has various useful applications, such as therapeutic, prophylactic, and growth promoters. However, the favorable effect of antibiotics is challenged by the risky development of resistance in human flora as well as pathogenic microbes and harmful chemical residues in meat (1). Probiotics dietary supplements in chickens are known to limit the usage of antibiotics and to improve the meat quality (2). Moreover, a hen's egg is an intensely nourishing product and a wealthy contributor to various pharmaceutical substances (3). Immunoglobulins (Igs) that are available in egg yolks were utilized on a large scale for research as well as clinical purposes, including the prevention of gastrointestinal infections. Maternal chicken Igs are passed to offspring through the egg yolk to provide passive immunization (4). The egg yolk IgY could likewise replace the natural generation of conventional polyclonal antibodies in mammals (5). Recently, we showed that the supplementation of purified IgY in combination with probiotics could remarkably improve the overall activity of broilers with immune stress; this effect was referred to the drop of immune cell count, which is responsible for inflammatory cytokine production and, consequently, the exaggerated stress during the innate immune response (6). Stressful conditions make birds more vulnerable to infection through the depletion of serum Igs and lowering the macrophage phagocytic ability, with a subsequent elevation in offal bacterial count (7). Moreover, immune stress prompts a decline in feed intake (FI), body weight, and digestibility and leads to an overall decline in growth performance and productivity (8). Regarding meat quality, immune stress could adversely impact meat quality through the rapid reduction of pH and quality traits, including the water holding capacity (WHC) (9). The improvement of meat quality traits related to appearance, such as color, is quite critical for product marketing and to the economic value of fresh meat. However, for marketing and purchase of cooked meat, the tenderness, palatability, and juiciness will gain much more importance (10, 11). Unacceptable color and tough broiler meat are most vital for their impact on consumer attitude and can cause a huge economic damage in the processing industry. The literature available on the contribution of IgY and probiotics combination to improve the meat quality and the nutritional values is scarce and should be emphasized. Therefore, the current study reveals the influence of feed additives, including the purified IgY powder, probiotics, and the combination of both of them, as an alternative to antibiotics, on the productivity of Ross broiler chicks and their meat quality. Moreover, the antimicrobial activity of the studied feed additives was investigated. The merit of this study is to produce high-quality organic meat from broilers that did not consume any chemical compounds during their life cycle.

## Materials and Methods

### Ethical Approval

During the whole period of the study (day 1 to 42), chick disturbance was kept at a minimal level in order to maintain their welfare. We rear the birds during appropriate weather conditions in the spring of 2019. The trials in the current study were performed following the regulations of the Animal Ethics Committee at the Faculty of Veterinary Medicine, South Valley University, Qena, Egypt. The experimental birds were caught, marked, and handled according to the committee license (161411-04-2018).

### Bird Management and Grouping

**Figure 1:**
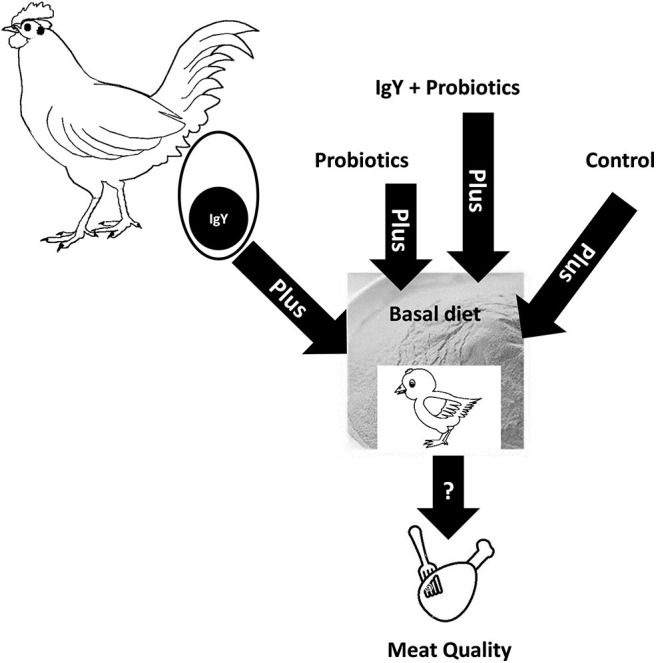
Schematic cartoon of the experimental strategy.

All essential sanitary requirements were performed in the poultry raising units. This experiment was performed on 40 female Ross chicks (1.2 ± 0.43 days old), which ranged from 48 to 50 g in weight and were purchased from Nutrivet Animal Health, Co., Ltd., Egypt. The chicks were separated into four groups (*n* = 10/group). The experimental birds were assessed for their performance, productivity, and carcass traits. The management and the vaccination protocols of the birds were done as previously described (1). All chicks were fed a commercial diet purchased from Alaaf Almagd, Alarabia Lell-alaaf, Quesna, Menofia, Egypt (12), besides *ad libitum* access to water. The basal diet was composed of yellow corn, soy bean meal, corn gluten, soy oil, dicalcium phosphate, lime stone, common salt, sodium bicarbonate, vitamins, minerals, choline chloride, DL-methionine, and L-lysine. The chemical composition of the diet is presented in Table S1 (2). The FI was recorded weekly. The experimental groups (shown in the schematic cartoon in Figure 1) were classified into (i) control group: chick broilers fed a basal diet, (ii) probiotics group: chick broilers supplemented with a probiotic mixture PRO-PAC® (Nutrivet Animal Health, Co., Ltd., Egypt) from day 1 to 42 of age, at 0.5 g/kg, (iii) IgY group: chick broilers supplemented with IgY powder (0.5 g/kg) which is added from day 8 to 42, and (iv) combination group: chick broilers supplemented with a mixture of both IgY and probiotics (0.25 g each per kilogram). Each kilogram of PRO-PAC® was composed of 0.1 kg of betaine HCl 97%, 0.1 kg of *Lactobacillus acidophilus*, 0.05 kg of *Enterococcus faecium*, 4.8 g of *Lactobacillus plantarum*, 2 g of *Bifidobacterium bifidum*, 0.05 kg of *Aspergillus oryzae* fermentation extracts (xylanase 12,500 units/kg, hemicellulase 2,750 units/kg, and ß-glucanase 2,250 units/kg), and 50 g/kg *Bacillus subtilus* fermentation extracts (α-amylase 25,000 units/kg, cellulose 4,500 units/kg, and protease 12,500 units/kg). We optimized the dose of PRO-PAC® and IgY powder based on our previous study (6), which improved the broilers' performance, physiological parameters, and productivities. PRO-BAC® was given to the birds from day 1 to maintain normal intestinal microflora and increase digestive enzyme activities (2, 6). However, the newly hatched chicks will be provided with egg yolk IgY until the end of their first week of life. Subsequently, the level of circulating IgY of chicks decreased considerably (4, 6), and therefore we decided to add IgY in the diet starting from day 8. With advancement of age of the chicks, the amount of probiotic and/or IgY powder will be increased based on the diet per kilogram introduced.

### IgY Preparation

The egg yolk precipitation was done using ammonium sulfate, and the fertile egg should contain up to 100 mg egg yolk (13). IgY was delivered carefully, using the water dilution technique, from the egg yolk as a cost-effective and simple technique to purify IgY from egg yolk. We used the cryoethanol method to obtain purified IgY because the ethanol concentration influenced the IgY recovery. Moreover, we have to adjust the temperature (−20°C) to avoid denaturing the proteins and to thoroughly remove ethanol. After salt precipitation, the cryoethanol treatment gave an IgY isolate of 96% yield and purity (14). Then, it was transformed to powder form by heating in order to stabilize the antibody molecule, as previously explained (15, 16).

### Antimicrobial Bioactivity Assay

The antimicrobial bioactivity assay was performed as mentioned before (17), using the liquid broth method. Moreover, *Staphylococcus aureus, Listeria monocytogenes, Escherichia coli*, and *Salmonella typhimurium* were generous gifts from the Bacteriology Unit, Reference Laboratory for Quality Control of Poultry Production, Animal Health Research Institute, El-Dokki, Giza, Egypt. Furthermore, incubation was done in the absence of protein (18). A mixture of an equal amount of the bacterial suspension and an equal volume of the tested sample (IgY and/or probiotic preparation) was made. This mixture was incubated, as previously described (19). The killing power of multiple treatments against bacteria was determined as log^10^ reduction in colony-forming units (CFU). Moreover, the result is presented as log CFU/ml, a function of (IgY and/or probiotic) concentration in the test medium as shown before (20).

### Broiler Performance and Health Status

To evaluate broiler performance and its health status, body weight, weight gain (WG), FI, feed conversion ratio (FCR), and culls have been registered on day 1 of age. Then, weekly record was done until day 42.

### Slaughtering and Carcass Yield

On day 42, the birds were weighed immediately before slaughtering by the decapitation technique of euthanasia. During euthanasia, caution was taken to reduce bird stress. The weights of the carcasses and their offal were assessed after slaughtering of the broilers (21). Manual evisceration and weighting of the carcass, heart, liver, and gizzard were performed. Then, the eviscerated carcasses were stored at −20°C for analysis.

### Analyses

#### Nutritive Value of Meat

A proximate analysis of broiler meat was achieved. Moisture was analyzed using oven drying (22), ash was determined using a muffle furnace (23), protein was analyzed using the Kjeldahl method (22), fat was determined using Soxhlet extraction (24), and thiobarbituric acid (TBA) was determined as explained in a previous method of Vyncke (25).

### Determination of Meat Quality

#### Measurement of pH

The pH was measured in the breast and the thigh muscles after 1 day of cooling storage of the carcass, using a pH meter, as described previously (26).

#### Meat Color Measurement

Color measurement was evaluated using a Chroma meter (Konica Minolta, model CR 410, Japan). It was calibrated with a white plate and a light trap and prepared by the producer (27). A total of three spectral readings were recorded for every meat sample. Lightness (L^*^) values [dark (0) to light (100)], redness (a^*^) values [reddish (+) to greenish (–)], and yellowness (b^*^) values [yellowish (+) to bluish (–)] were measured.

### Determination of Cooking Loss

Cooking loss was determined through the technique stated before (21), using the following equation: cooking loss (%) = [(*F* – *G*)/(*F*)] × 100, where *F*, weight of the uncooked sample and *G*, weight of the cooked sample.

### Shear Force Measurement

Instron Universal Testing Machine (model 2519-105, USA) was used to determine the shearing force of the cooked meat. To assess the shear force, 2.0 cm^2^ from cooked breast meat was utilized, using a crosshead speed of 200 mm/min (28).

### Determination of Water Holding Capacity

WHC was determined as described before (29), using the following calculation:

(1) $$\text{WHC=}\frac{\text{Weight after pressing}}{\text{Weight before pressing}}\times \text{100}$$

### Microbiological Analysis of Meat

Ten grams of meat sample was added to 90 ml of saline. The sample was homogenized for 10 min at 3,000 rpm with a sterile blender. Then, serial dilutions were done for the microbiological analysis (30). The sanitary status of the meat was evaluated by aerobic bacteria count using plate count agar, coliform count using violet red bile agar, *S. aureus* count using mannitol salt agar, and *E. coli* count using eosin methylene blue agar (31).

### Estimation of Mortalities

Daily mortality rates in the control and the treated groups were recorded.

#### Economic Impact

Calculation of the cost (per $) of chicken meat (per kilogram) and the nutritive value for each treatment was done at day 42.

### Statistical Analysis

Statistics were conducted using the SPSS statistical system (version 16). The data were obtained using duplicates, the analysis was done using one-way ANOVA, and the comparison of averages was carried out using Duncan's multiple-range tests. Moreover, the data were stated as mean ± SEM, and the differences were significant at *P* < 0.05. The *F* distribution has two parameters, the between-groups degrees of freedom, *k*, and the residual degrees of freedom, *N* – *k*, represented as the following ANOVA formula: df1 = *k* – 1, df2 = *N* – *k*), where df, degrees of freedom; *k*, number of groups; *N*, number of observations.

## Results

### Determination of Antimicrobial Bioactivity of IgY, Probiotics, and IgY/Probiotic Group

**Figure 2:**
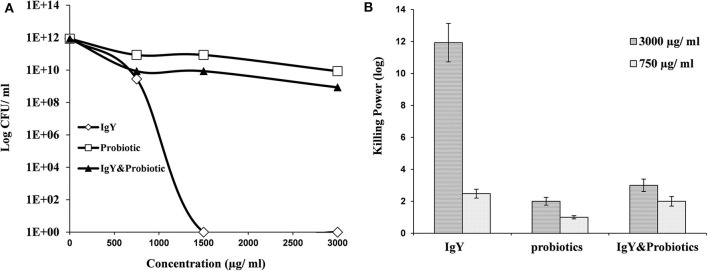
**(A)** Antibacterial activity of IgY, probiotic, and the mixture of both, at different concentrations, against *Staphylococcus aureus*. **(B)** Log^10^ of the antibacterial activity for the same groups at two concentrations (750 and 3,000 μg/ml) against *S. aureus*.

**Figure 3:**
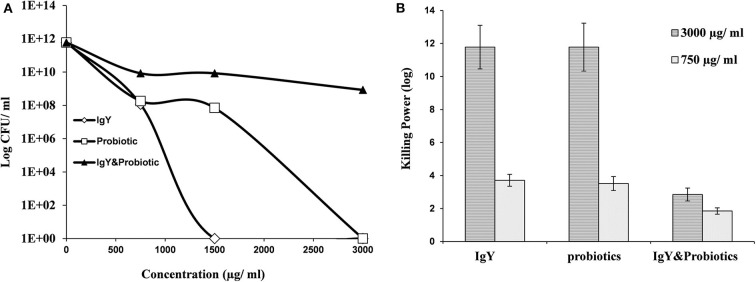
**(A)** Antibacterial activity of IgY, probiotic, and the mixture of both, at different concentrations, against *Listeria monocytogenes*. **(B)** Log^10^ of the antibacterial activity for the same groups at two concentrations (750 and 3,000 μg/ml against *L. monocytogenes*).

Interestingly, *S. aureus* and *E. coli* could not be detected in the meat samples of probiotic and combination broiler groups, respectively (shown in Table 1). This might be a result of the killing power shown by probiotics and IgY against *S. aureus* (Figure 2A) and achieved consequently at both higher and lower concentrations (3,000 and 750 μg/ml) (Figure S1B). Moreover, IgY showed evidence of killing power (log CFU/ml) against *S. aureus* and *L. monocytogenes* at a concentration of 1,500 μg/ml (Figures 2A, 3A). However, probiotics killed *L. monocytogenes* at 3,000 μg/ml, but there were no effects shown on *S. aureus* (Figures 2B, 3B, respectively). On the contrary, the concentration of IgY powder used has no killing power against gram-negative bacteria, including *E. coli* (Figures S1A,B) and *S. typhimurium* (Figures S2A,B). The result revealed the efficient antimicrobial bioactivity of IgY powder in *in vitro* as well as *in vivo* studies.

**Table 1:** Mean bacterial count in broilers meat of the different experimental groups.

**No**.	**Treatment**	**Aerobic bacteria**	***Staphylococcus aureus***	***Escherichia coli***
1	CNT	4.25E4	4.33E3	1.50E3
2	Probiotic	1.50E4	–	5.00E3
3	IgY	5.65E4	4.90E3	5.00E2
4	Probiotic + IgY	2.98E4	7.40E3	–

*CNT, control group; IgY, immunoglobulin Y; –, not detected*.

### Determination of the Health and the Growth Rates of Ross Broilers After Feed Additive Supplementation

The combination group has increased average WG in the starter to the grower phase of birds, from week 2 (219.6 ± 8.6 g) to week 4 (483.6 ± 10.06 g) [*F*_(3, 36)_ = 2.452 and *F*_(3, 36)_ = 2.534, *P* = 0.05 each] than in controls (166 ± 21.2 and 429 ± 24.8 g, respectively) (Table S2). Moreover, FI was the highest at week 6 (961.3 ± 134.1 g) in the IgY group, followed by the combination group (952.6 ± 122.7 g), compared to the control (900 ± 115.2 g) (see Table S3). The results in Table 2 showed that the combination group reached the peak of the birds' weights at week 2–6 as 355 ± 25.3, 743.0 ± 74.8, 1,226.6 ± 112.3, 1,747.1 ± 134.5, and 2,253.5 ± 143.9 g, respectively. Therefore, the weights of birds in those weeks achieved significant differences [*F*_(3, 36)_ = 2.324, *P* = 0.041; *F*_(3, 36)_ = 2.362, *P* = 0.032; *F*_(3, 36)_ = 2.423, *P* = 0.022; *F*_(3, 36)_ = 2.443, *P* = 0.025; and *F*_(3, 36)_ = 2.468, *P* = 0.037], compared to the controls. Consequently, the FCR was altered in broilers with aging. For instance, the IgY group revealed the highest FCR at the grower phase (1.36% at week 3); the IgY and combination groups also had high FCRs at the finisher phase (1.8% each) at week 6 (Table S4). Our results confirmed that the appetite and the growth rate of the broilers have been improved after feed supplementation with probiotics and IgY.

**Table 2:** The statistics of weights (g) of broilers supplemented with variant feed additives.

**No**.	**Group**	**W (week-1)**	**W (week-2)**	**W (week-3)**	**W (week-4)**	**W (week-5)**	**W (week-6)**
1	CNT	135.6 ± 3.7	301.6 ± 22.9	677.3 ± 93.5	*1, 106.3*±169.7	*1, 609.3*±249.2	*2, 099.6*±323.5
2	Probiotic	130.3 ± 3.5	334 ± 13.02	732.6 ± 51.2	*1, 155*±79.05	*1, 653.2*±91.8	*2, 166.6*±143.2
3	IgY	131.6 ± 5.6	311.3 ± 12.6	700.3 ± 74.9	*1, 167.3*±112.8	*1, 654*±145.02	*2, 160.2*±183.05
4	Probiotic + IgY	135.3 ± 4.9	355 ± 25.3^*^	743 ± 74.8^*^	*1, 226.6*±112.3^*^	*1, 747.1*±134.5^*^	*2, 253.5*±143.9^*^

*The result was presented as mean ± SEM and analyzed using one-way ANOVA*.

*W, weight; CNT, control group; IgY, immunoglobulin Y*.

* *P < 0.05*.

### Impacts of Feed Additives on Carcass Characteristics and Meat Quality

The result in Table 3 showed a significant increase of carcass weight with viscera [2,095 ± 149.2 g, *F*_(3, 36)_ = 2.642, *P* = 0.006] and without viscera (1,753.3 ± 133.3 g) in the combination group. However, the IgY group had the highest weight of heart, liver, and gizzard (8.91 ± 0.4 g “*F*_(3, 36)_ = 2.324, *P* = 0.004,” 38.7 ± 1.4 g “*F*_(3, 36)_ = 2.326, *P* = 0.032,” and 38.92 ± 3.2 g “*F*_(3, 36)_ = 2.369, *P* = 0.042,” respectively). The best pH value of breast muscle was obtained in the probiotics group (5.71 ± 0.01), followed by the combination group [5.77 ± 0.05, *F*_(3, 36)_ = 2.388, *P* = 0.03]. The degree of redness was more acceptable in the combination group and the IgY group. Furthermore, yellowness was improved in the probiotics group (13.21 ± 0.12). Hence, supplementation of probiotic and IgY could improve the color of broiler meat to be more acceptable. The results in Table 4 show the average shearing force/compressive load as 2.20 ± 0.24, 3.34 ± 0.51, 2.43 ± 0.49, and 3.94 ± 0.01 kgf/cm^3^ in the control, probiotic, IgY, and combination groups, respectively. The result revealed that the WHC in the IgY group was the highest (21.97%), but the lowest (28.040%) in terms of cooking loss. It means that the tenderness of IgY was almost the same to that of normal broiler meat. Moreover, the IgY group was the most acceptable treatment with regard to WHC and cooking loss. Therefore, probiotics and IgY supplementation improved the carcass quality traits of broilers.

**Table 3:** The statistics of carcass and internal organ weights (g) of broilers supplemented with variant feed additives.

**No**.	**Group**	**W of carcass + viscera**	**W of carcass**	**W of heart**	**W of liver**	**W of spleen**	**W of gizzard**
1	CNT	*1, 731.6*±12.02	*1, 443.3*±15.89	7.14 ± 0.3	32.95 ± 0.96	1.57 ± 0.12	35.26 ± 1.79
2	Probiotic	*1, 835*±136.1	*1, 528.3*±131.41	8.73 ± 0.02	36.08 ± 3.34	2.26 ± 0.49	31.95 ± 3.12
3	IgY	*2, 086.6*±25.2	*1, 758.3*±23.3	8.91 ± 0.4^**^	38.7 ± 1.4^*^	1.79 ± 0.2	38.92 ± 3.2^*^
4	Probiotic + IgY	*2, 095*±149.2^**^	*1, 753.3*±133.3	8.49 ± 0.3	38.61 ± 0.9	1.32 ± 0.09	33.77 ± 2.3

*The result was presented as mean ± SEM and analyzed using one-way ANOVA*.

*W, weight; CNT, control group; IgY, immunoglobulin Y*.

* *P < 0.05*,

** *P < 0.01*.

**Table 4:** pH values of breast and thigh, color, shear force (kgf), water holding capacity percentage, and cooking loss percentage of broilers groups supplemented by variant feed additives.

**No**.	**Group**	**pH**		**Color**			**Shear force**	**WHC (%)**	**Cooking loss (%)**
		**Breast**	**Thigh**	L*	a*	b*	**(kgf)**		
1	CNT	5.79 ± 0.08	6.12 ± 0.06	56.87 ± 1.78	9.63 ± 1.02	15.88 ± 0.27	2.20 ± 0.24	29.28	24.2
2	Probiotic	5.71 ± 0.01^*^	6.18 ± 0.09	58.01 ± 0.45	8.92 ± 0.02	13.21 ± 0.12	3.34 ± 0.51	17.61	32.215
3	IgY	5.89 ± 0.1^**^	6.24 ± 0.06	60.97 ± 1.09	7.85 ± 3.04	17.30 ± 1.37	2.43 ± 0.49	21.97	28.04
4	Probiotic + IgY	5.775 ± 0.05^*^	6.115 ± 0.06	60.0 ± 0.01	7.67 ± 0.01	17.80 ± 0.01	3.94 ± 0.01	19.67	31.45

*The result was presented as mean ± SEM and analyzed using one-way ANOVA*.

CNT, control group; IgY, immunoglobulin Y, kgf, kilogram-force; WHC, water holding capacity; L*, Lightness; a*, redness; b*, yellowness.

* *P < 0.05*,

** *P < 0.01*.

### Impact of Feed Additives on the Nutritive Value of Meat

As shown in Table 5, the protein percentage was the lowest (18.9 ± 0.18) in the controls. However, the protein percentage in the combination group [22.2 ± 0.29, *F*_(3, 36)_ = 2.367, *P* = 0.001] was the highest record. The control group recorded the highest in moisture percentage (77 ± 0.34); however, the probiotics group was the lowest (74.69 ± 2.6). Ash percentage was the highest in the latest group [1.69 ± 0.03, *F*_(3, 36)_ = 2.391, *P* = 0.005]. The probiotic and combination groups had significantly increased protein and ash content in the meat, respectively. Moreover, the TBA value was reduced in the IgY (0.24 ± 0.06) and the combination groups (0.16 ± 0.01). Therefore, feed supplemented with IgY and/or probiotics improved the nutritive value of broiler meat.

**Table 5:** Nutritive and TBA values of broiler meat in the experimental groups.

**No**	**Treatment**	**Nutritive value**				**TBA value (mg malondialdehyde/kg)**
		**Protein %**	**Ash %**	**Moisture %**	**Fat %**	
1	CNT	18.93 ± 0.185	1.23 ± 0.059	77.00 ± 0.34	2.05 ± 0.06	0.243 ± 0.017
2	Probiotic	22.13 ± 0.37	1.69 ± 0.03^**^	75.72 ± 0.82	2.20 ± 0.16	0.274 ± 0.02
3	IgY	19.0 ± 0.77	1.09 ± 0.007	76.92 ± 0.22	2.18 ± 0.10	0.242 ± 0.06
4	Probiotic + IgY	22.26 ± 0.29^***^	1.09 ± 0.007	76.62 ± 0.41	2.29 ± 0.05	0.167 ± 0.01

*The result was represented as mean ± SEM and analyzed using one-way ANOVA*.

*CNT, control group; IgY, immunoglobulin Y; TBA, thiobarbituric acid*.

** *P < 0.01*,

*** *P < 0.001*.

### Impact of Feed Additives on the Economic Benefits of Poultry Farms

Our result showed no mortalities in the broiler groups of varied feed supplementations, compared to the controls, (Table S5). Surprisingly, the highest weight (2,253.5 ± 143.9 g) of a bird at week 6 was recorded in the combination group after a consumption of 3,415 g ration and supplementation with probiotic and IgY (0.85 g each) (Table 2 and Table S6). Economically, the net profit differences per bird were recorded for all groups (Table S7), and therefore we could confirm that the group of combined probiotics and IgY supplementation achieved the highest economic values ($0.76) along the broiler production cycle.

## Discussion

The literature handling the impact of IgY and probiotics combination on meat quality and antibacterial activity is scarce. Several reports discussed IgY stability, an antibacterial activity *in vitro* (32), even though there is limited research conducted *in vivo* to evaluate the capability of hens' egg antibodies in combating intestinal pathogenic bacteria in poultry farms. Herein we showed that *S. aureus* and *E. coli* could not be detected in the broiler meat of the probiotic and combination groups, which suggests the enhancement of the microbiological profile of meat by probiotic and IgY supplementation in poultry diet.

The determination of antimicrobial activities of either experimented IgY or probiotic (500 μl each) and a mixture of both (250 μl each) *in vitro* revealed that IgY can also retain a bactericidal activity against gram-positive bacteria including *S. aureus* and *L. monocytogenes* at a concentration of 1,500 μg/ml. These findings coincided with a previous report that showed an effective bactericidal activity of IgY against *S. aureus* growth, suggesting the therapeutic benefits of IgY (33). This result was also consistent with a previous study (34) that showed the ability of IgY to suppress *Staphylococcus* in chicken meat. Furthermore, we showed here that probiotics can efficiently combat *L. monocytogenes* at 3,000 μg/ml, suggesting a broader scope of its bactericidal activity. However, no bactericidal activity was obtained from the mixture of IgY and probiotics *in vitro*. This effect might be a result of the antagonism between bactericidal and bacteriostatic products (35). Therefore, the combination of probiotics, which own bacteriostatic activities, with IgY might be preferably done during ration formulation to maximize their beneficial effect (6). However, we confirmed here that egg yolk-derived IgY, as a bactericidal product with various maternal specific antibodies, has more sensitivity against gram-positive bacteria. However, the limitation of the killing power of IgY against gram-negative bacteria is probably due to its genetic resistance and/or its outer membrane. Further experiments are required to optimize the effective concentration or make some combinations with other substances to disrupt the outer membranes.

We fed the chickens a basal diet, probiotics, IgY powder, or IgY/probiotics combination to address the influence on meat quality, immunity performance, and productivity. The FI was the highest at week 6 in the IgY group, followed by the combination group compared to the control birds. Similar to previous results (36), an increase in the FI of broilers was obtained from using various strains of *Saccharomyces cerevisiae* probiotics in comparison to the controls. Consequently, FI might contribute to high FCR in healthy birds. High FI is more efficient with the proteolytic stability of IgY in the stomach and intestines. We showed that the combination group had reached the peak of their weights at week 2 (starter phase) and week 6 (finisher phase). Our findings were consistent with the reports showing that supplementing broiler with yeast probiotics increased their weight gain (37). Feeding birds with IgY and/or probiotic can likely improve their FCRs during the production cycle (38). Therefore, our findings revealed a positive relationship between FI and bird performance.

The weights of the carcass with viscera or without viscera increased in the combination group. Also, the IgY group had the highest weights of heart, liver, and gizzard. Our results coincided with a previous report showing a significant difference in the live weight gain of the group fed with probiotics compared to the other one fed a basal diet (39). So, the weights of carcasses were significantly changed according to the nature of feed additives.

The pH values for breast muscle in the probiotics and combination groups (*P* < 0.05 each) were more acceptable in comparison to that of the controls. Moreover, supplementation with IgY improved the bird's activities and performance (6), which leads to a decrease in glycogen storage in muscles and consequently limitation of lactic acid amount in post-mortem glycolysis. Therefore, it might explain the reason of the less acidic pH of the meat in the IgY group (*P* < 0.01) compared to that of controls. Besides that, the pH of the thigh in the combination group was more acceptable, with no significance. Hence, the storage quality of these groups was improved. This may influence the tenderness and the organoleptic and functional characteristics of raw and processed products. Moreover, the decreased pH is an unfavorable medium for the action of spoilage microorganisms.

It is known that the color of raw poultry meat is a crucial quality trait of fresh meat. Therefore, we used an average of spectral reading to detect the lightness, redness, and yellowness of the meat for each bird. The lightness (L^*^) in all groups was more than the normal value (L^*^ = 53), as reported by a previous study (40, 41). We also stated that the degree of redness was more acceptable in the IgY group, followed by the combination group, indicating that the IgY and probiotics combination improved the a^*^ value. Furthermore, yellowness was represented by b^*^ values, which showed a more acceptable value in the probiotics group. Our findings are in line with a previous study (42) that reported an improvement in the yellowness values (b^*^) of broiler meat obtained by feeding *Lactobacillus salivarius* probiotics. The results obtained matched a reported hypothesis that pH has a negative correlation to L^*^ values. For instance, it was stated that a significantly lighter chicken muscle had a lower pH (43). Moreover, the elevation of meat pH was closely linked to dark muscle pigmentation than lower pH (44). Therefore, dietary probiotics and IgY enhanced the quality of broiler meat by improving the pH and color values (45).

Meat tenderness was known as the main quality characteristic considered for consumer acceptability and purchasing of meat. The obtained findings showed that feeding IgY is the best treatment as it produces tender meat. WHC can be defined as the capability of the meat to retain its intrinsic moisture, all or part of its water, even with external pressure. Drip loss, WHC, and cooking loss were determined as a general evaluation of the water-binding characteristics of meat. Meanwhile, cooking loss is a significant feature for the processing sector since water retention is a major gain point (40). In the current work, the WHC percentage was higher in IgY (21.97%), compared to a percentage of 19.67% in the combination group and 17.61% in the probiotics group. Therefore, the IgY group was the lowest (28.04%) in cooking loss. We can conclude that IgY is a more acceptable treatment among all since it has a strong ability to hold water and lower cooking loss.

Concerning the nutritive value, the protein percentage was significantly highest in the combination group. The highest percentage of moisture was also recorded in the controls, while the lowest percentage was obtained in the probiotics group, indicating the possible increased shelf-life for broiler meat in these groups. Hence, moisture is considered a good medium for the multiplication of spoilage bacteria; we supposed that the low moisture content of meat in the probiotics group would improve the product durability. The percentage of ash in meat was the highest in the probiotics group. Fat oxidation is the main reason of the rancidity and the poor flavor in the meat. Malondialdehyde is a common aldehyde produced during fat oxidation. Malondialdehyde is an extremely toxic compound that can disrupt a variety of physiological functions in man. In the current work, the TBA assay was used to determine malondialdehyde. The TBA value was reduced in the combination groups followed by the IgY group, indicating the more stability of fat and greater enhancement to the meat shelf-life. It was revealed that the meat of the IgY group showed a significant reduction of TBA values during storage compared to those of the controls (45). Our results were in line with a previous report (46) which states that the TBA value of breast meat in broiler fed with *B. subtilis* was 0.20 mg (malondialdehyde/kg meat), compared to the values of the non-probiotics category.

Our results indicated no mortalities in birds fed IgY, which agreed with the authors who stated that egg yolk antibodies can prevent fatal *Salmonellosis* in newly born offspring and maintain their life (47). Moreover, the probiotics improved the bird's performance and health (2, 6, 28, 38). The highest weight of birds at week 6 was determined in the combination group. The average of the broiler's economic gain difference was also at its peak in the combination group ($0.76), followed by the probiotics group ($0.73). However, the control group recorded mortalities, which influenced the FI and therefore had the lowest economics ($0.43). Moreover, the chemical, physical, and microbiological analyses of meat should be regulated by the behavior and the physiological status of broilers, particularly the group supplemented with IgY. Therefore, IgY is beneficial for enhancing immunity and meat safety, maximizing production, increasing profitability, and improving the welfare of broilers.

## Conclusion

The present work presented the outcomes of different feed supplementations on the growth performance and meat quality of broilers. The combination of IgY and probiotics in the feeds of broilers is the best choice for maximizing meat production, providing better immune status, and improving the carcass quality and economic benefits. Therefore, using IgY with probiotic in large-scale production technology, as a cost-effective feed additive in poultry farms as well as for producing organic meat, is strongly recommend. The consequences of the present work provide interesting clues for further exploration of the communication between probiotics and IgY additives and their influence on the gut microbial diversity and the main fermentation products in the digestive system of broilers.

## Data Availability Statement

The raw data supporting the conclusions of this article will be made available by the authors, without undue reservation.

## Ethics Statement

The animal study was reviewed and approved by Animal Ethics Committee at the Faculty of Veterinary Medicine, South Valley University, Qena, Egypt.

## Author Contributions

MH, IR, AR, NE, and MM mutually contributed to the hypothesis and the design of the scientific manuscript. AR, MM, MY, MH, NE, and MA-R provided the chemicals and the materials used in this work. AR, IR, MM, SF, AA, MY, HD, GB, SA, HK, OS, EA, HH, AEln, AElk, and AH performed the experimental procedures and analysis. All authors participated in this research work. They also participated in writing the manuscript's draft and revision. All authors have read and approved the final manuscript. The authors agreed to publish the findings generated from this work.

## Conflict of Interest

The authors declare that the research was conducted in the absence of any commercial or financial relationships that could be construed as a potential conflict of interest.

## Abbreviations

IgY: immunoglobulin Y
L^*^: lightness
a^*^: redness
b^*^: yellowness
TBA: thiobarbituric acid
WHC: water holding capacity
FI: feed intake
WG: weight gain
FCR: feed conversion ratio.
