# Supplementary material for: Egg Yolk IgY: A Novel Trend of Feed Additives to Limit Drugs and to Improve Poultry Meat Quality
Source: Front Vet Sci. 2020 Jul 14;7:350. doi: 10.3389/fvets.2020.00350 (PMC7371932; doi:10.3389/fvets.2020.00350)
Supplement: Supplementary file 1 [file Data_Sheet_1.docx]

**SUPPLEMENTARY MATERIAL**

**Egg Yolk IgY: A novel Trend of Feed Additives to Limit Drugs and to Improve Poultry Meat Quality**

**Running Title: IgY-technology Improves the Meat Quality**

***Mohamed A. Hussein^1^, Ibrahim F. Rehan^2*^, Ahmed F. Rehan^1^, Nesreen Z. Eleiwa^3^, Mootaz A. M. Abdel-Rahman^4^, Sohaila G. Fahmy^5^, Ahmed S. Ahmed^6^, Mohammed Youssef^7^, Hassan M. Diab^8^, Gaber E. Batiha^9^, Sara T. Alrashood^10^, Haseeb A. Khan^11^, Obeid Shanab^12^, Eslam Ahmed^5^, Hamdy Hassan^13^, Asmaa Elnagar^14^, Amr Elkelish^15^, Abd El-Latif Hesham^16^and Mohamed A. Maky^17*^***

^1^ Department of Food Control, Faculty of Veterinary Medicine, Zagazig University, Zagazig, Egypt.

^2^ Department of Husbandry and Development of Animal Wealth, Faculty of Veterinary Medicine, Menofia University, Shebin Alkom, Menofia, Egypt.

^3^ Department of Food Hygiene, Animal Health Research Institute, Agricultural Research Center, Giza, Egypt.

^4^ Department of Behavior, Management and Development of Animal Wealth, Faculty of Veterinary Medicine, Minia University, El-Minia, Egypt.

^5^ Department of Animal Behaviour and Management, Faculty of Veterinary Medicine, South Valley University, Qena, Egypt.

^6^ Department of Food Hygiene and Control (Milk Hygiene), Faculty of Veterinary Medicine, South Valley University, Qena, Egypt.

^7^ Department of Animal Physiology, Faculty of Veterinary Medicine, South Valley University, Qena, Egypt.

^8^ Department of Animal and Poultry Health and Environment, Faculty of Veterinary Medicine, South Valley University, Qena, Egypt.

^9^ Department of Pharmacology and Therapeutics, Faculty of Veterinary Medicine, Damanhour University, Damanhour, Al Beheira, Egypt.

^10^ Department of Pharmaceutical Chemistry, College of Pharmacy, King Saud University, Riyadh, Saudi Arabia.

^11^ Department of Biochemistry, College of Science, King Saud University, Riyadh, Saudi Arabia.

^12^ Department of Biochemistry, Faculty of Veterinary Medicine, South Valley University, Qena, Egypt.

^13^ Department of Animal Production, Faculty of Agriculture, South Valley University, Qena, Egypt.

^14^ Department of Biochemistry, Faculty of Veterinary Medicine, Zagazig University, Zagazig, Egypt.

^15^ Department of Botany, Faculty of Science, Suez Canal University, Ismailia, Egypt.

^16^ Department of Genetics, Faculty of Agriculture, Beni-Suef University, Beni-Suef, Egypt.

^17^ Department of Food Hygiene and Control (Meat Hygiene), Faculty of Veterinary Medicine, South Valley University, Qena, Egypt.

***Corresponding authors**: Ibrahim F. Rehan ([ibrahim.rehan@vet.menofia.edu.eg](mailto:ibrahim.rehan@vet.menofia.edu.eg)) and Mohamed A. Maky ([mohamedmekky@vet.svu.edu.eg](mailto:mohamedmekky@vet.svu.edu.eg)).

**TABLE S1** The chemical compostion of the diet.

| **Ingredients** | **Ration** | | |
| --- | --- | --- | --- |
|  | **Starter** | **Grower** | **Finisher** |
| **Crude protein (%)** | 23 | 21 | 19 |
| **Crude fat (%)** | 5.92 | 6.62 | 6.86 |
| **ME (kcal/kg)** | 3020 | 3100 | 3200 |
| **Crude fiber (%)** | 3.76 | 3.46 | 3.2 |

**TABLE S2** The statistics of weights gain (g) of chick broiler supplemented with variant feed additives.

| **No.** | **Group** | **WG**  **(week-1)** | **WG**  **(week-2)** | **WG**  **(week-3)** | **WG**  **(week-4)** | **WG (week-5)** | **WG**  **(week-6)** |
| --- | --- | --- | --- | --- | --- | --- | --- |
| **1** | **CNT** | 86.3 ± 5.9 | 166 ± 21.2 | 375.7 ± 24.2 | 429 ± 24.8 | 503 ± 14.8 | 490.3 ± 26.4 |
| **2** | **Probiotic** | 84.6 ± 2.7 | 203.7 ± 17.06 | 398.6 ± 11.6 | 422.4± 10.5 | 498.2± 28.4 | 513.4 ± 16.3 |
| **3** | **IgY** | 87 ± 6.07 | 179.7 ± 13.1 | 389 ± 11.8 | 467± 26.7 | 486.7 ± 61.9 | 506.2 ± 64.2 |
| **4** | **Probiotic + IgY** | 86.6 ± 4.2 | 219.7 ± 8.6* | 388 ± 16.7 | 483.6 ± 10.06* | 520.5 ± 62.6 | 506.4 ± 36.5 |
| *The result was presented as mean ± SEM and analyzed using one-way ANOVA. WG: weight gain; CNT: control group; IgY: immunoglobulin Y; (*), P < 0.05.* | | | | | | | |

**TABLE S3** The mean values of feed intake (g) of chick broiler supplemented with variant feed additives.

| **No.** | **Group** | **FI (week-1)** | **FI (week-2)** | **FI (week-3)** | **FI (week-4)** | **FI (week-5)** | **FI (week-6)** |
| --- | --- | --- | --- | --- | --- | --- | --- |
| **1** | **CNT** | 181 ± 22.1 | 340 ± 42.2 | 502.3 ± 74.3 | 569.3 ± 75.4 | 906 ± 135.2 | 900 ± 115.2 |
| **2** | **Probiotic** | 180.6 ± 19.3 | 349.3 ± 44.1 | 508.3 ± 76.4 | 572.6 ± 77.1 | 842.3 ± 116.3 | 904.6 ± 117.3 |
| **3** | **IgY** | 176.6 ± 15.7 | 356.6 ± 50.2 | 531.3 ± 74.1 | 604.3 ± 88.2 | 875.3 ± 122.4 | 961.3 ± 134.1 |
| **4** | **Probiotic + IgY** | 182.3 ± 22.4 | 356.6 ± 51.4 | 482.6 ± 66.7 | 610 ± 90.3 | 831 ± 111.7 | 952.6 ± 122.7 |
| *The result was presented as mean ± SEM and analyzed using one-way ANOVA. FI: feed intake; CNT: control group; IgY: immunoglobulin Y.* | | | | | | | |

**TABLE S4** The feed conversion ratio of chick broiler supplemented with variant feed additives.

| **No.** | **Group** | **FCR** | **FCR** | **FCR** | **FCR** | **FCR** | **FCR** |
| --- | --- | --- | --- | --- | --- | --- | --- |
|  |  | **(week-1)** | **(week-2)** | **(week-3)** | **(week-4)** | **(week-5)** | **(week-6)** |
| **1** | **CNT** | 2.09 ± 0.21 | 2.04 ± 0.16 | 1.33 ± 0.1 | 1.32 ± 0.11 | 1.8 ± 0.15 | 1.83 ± 0.11 |
| **2** | **Probiotic** | 2.13 ± 0.17 | 1.71 ± 0.12 | 1.27 ± 0.09 | 1.35 ± 0.09 | 1.69 ± 0.13 | 1.76 ± 0.1 |
| **3** | **IgY** | 2.02 ± 0.15 | 1.98 ± 0.14 | 1.36 ± 0.05 | 1.29 ± 0.08 | 1.79 ± 0.12 | 1.89 ± 0.12 |
| **4** | **Probiotic + IgY** | 2.1 ± 0.16 | 1.62 ± 0.11 | 1.24 ±0.09 | 1.26 ± 0.09 | 1.59 ± 0.11 | 1.88 ± 0.13 |
| *The result was presented as mean ± SEM and analyzed using one-way ANOVA. FCR: feed conversion ratio; CNT: control group; IgY: immunoglobulin Y.* | | | | | | | |

**TABLE S5** Calculation of mortality percentage of chicken broilers (n=10) during the experiment.

| **No.** | **Group** | **Day** | **Dead** | **Mortality %** |
| --- | --- | --- | --- | --- |
| **1** | **Control** | 7 | 1 | 20 |
|  |  | 34 | 1 |  |
| **2** | **Probiotic** | ─ | ─ | 0 |
| **3** | **IgY** | ─ | ─ | 0 |
| **4** | **Probiotic + IgY** | ─ | ─ | 0 |
| *IgY: immunoglobulin. (─): not detected.* | | | | |

**TABLE S6** Average weights (g) of ration and feed additive (n=10) consumed per bird in the experiment.

| **No.** | **Group** | **W (g)/ bird** | |
| --- | --- | --- | --- |
|  |  | **Ration** | **Feed additive** |
| **1** | **Control** | 3398.6 | ─ |
| **2** | **Probiotic** | 3357.7 | 1.68 |
| **3** | **IgY** | 3505.4 | 1.75 |
| **4** | **Probiotic + IgY** | 3415.1 | 0.85 + 0.85 |
| *IgY: immunoglobulin; W: weight. (─): not given.* | | | |

**TABLE S7** Effect of various feed additives on economic values ($) of broiler farms in experimental groups.

| **No.** | **Group** | **Cost/bird ($)** | | | | | | | | | **Total cost/bird** | **Total return/bird** | | **Net profit/bird** |
| --- | --- | --- | --- | --- | --- | --- | --- | --- | --- | --- | --- | --- | --- | --- |
|  |  | **Chick** | **Ration** | **Feed additives preparation** | **Drugs** | **Vaccines** | **Disinfectant** | **Bedding** | **Supervision** | **Building** |  | **Poultry sale** | **Litter sale** | **(Total return-Total cost)** |
|  |  |  |  |  |  |  |  |  |  | **& equipment** | **($)** | **($)** | **($)** | **($)** |
| **1** | **Control** | 0.24 | 1.33 | ─ | 0.18 | 0.06 | 0.015 | free | 0.03 | free | 1.855 | 2.263 | 0.018 | (2.281-1.855) = 0.43 |
| **2** | **Probiotic** | 0.24 | 1.28 | 0.00078 | ─ | 0.06 | 0.015 | free | 0.03 | free | 1.626 | 2.335 | 0.018 | (2.353-1.626) = 0.73 |
| **3** | **IgY** | 0.24 | 1.37 | 0.0013 | ─ | 0.06 | 0.015 | free | 0.03 | free | 1.716 | 2.328 | 0.018 | (2.346-1.716) = 0.63 |
| **4** | **Probiotic + IgY** | 0.24 | 1.34 | 0.0004+0.00063 | ─ | 0.06 | 0.015 | free | 0.03 | free | 1.686 | 2.429 | 0.018 | (2.447-1.686) = 0.76 |
| *The data was obtained in Egypt on 2019. IgY: immunoglobulin;* *$: U.S dollar; free: freely available at the faculty farm. (* ─*): not given.We have to notice that the price of 1kg ration costed 0.39$, 1kg probiotic costed 0.47$ and 1kg IgY costed 0.75$ so the calculation in this table was estimated accodingly (e.g., 1 U.S. Dollar =17.80 Egyptian pound).* | | | | | | | | | | | | | | |

**
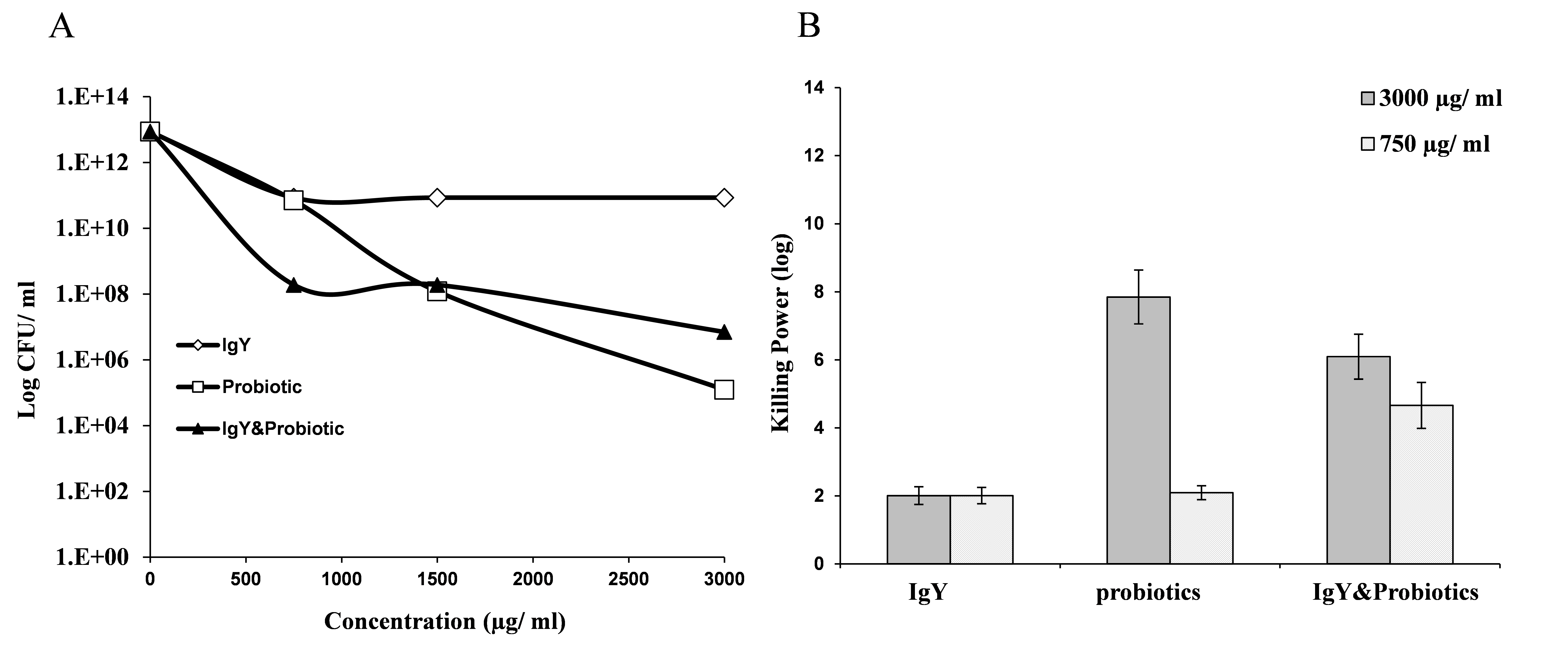
**

**FIGURE S1 (A)** Antibacterial activity of IgY, probiotic and the mixture of both against Escherichia coli at different concentrations; **(B)** Log^10^ of antibacterial activity for the same groups at two concentration of 750 µg/ ml and 3000 µg/ ml against E.coli.

**
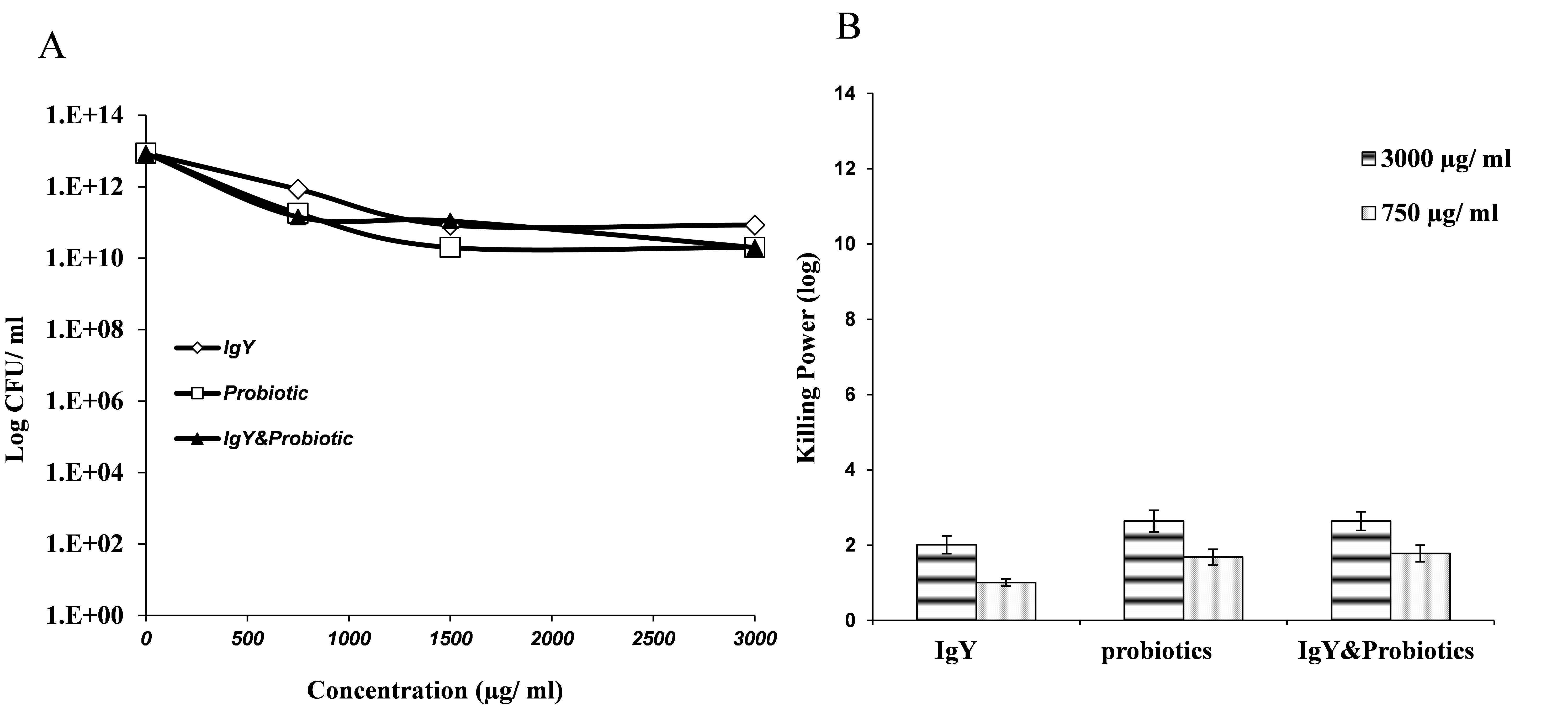
FIGURE S2 (A)** Antibacterial activity of IgY, probiotic and the mixture of both against Salmonella typhimurium at different concentrations; **(B)** Log^10^ of antibacterial activity for the same groups at two concentration of 750 µg/ ml and 3000 µg/ ml against S.typhimurium.
